# Study of The Effects of N-Acetylcysteine on Oxidative Stress Status of Patients on Maintenance-Hemodialysis Undergoing Cadaveric Kidney Transplantation

**Published:** 2017

**Authors:** Atieh Modarresi, Shadi Ziaie, Jamshid Salamzadeh, Zahra Sahraei, Mohsen Nafar, Yunes Panahi, Mahmoud Parvin, Behzad Einollahi

**Affiliations:** a *Department of Clinical Pharmacy, School of Pharmacy, Shahid Beheshti University of Medical Sciences, Tehran, Iran. *; b *Shahid Labbafinejad Medical Center, Urology and Nephrology Research Center, Shahid Beheshti University of Medical Sciences, Tehran, Iran.*; c *Pharmacotherapy Department, Faculty of Pharmacy, Baqiyatallah University of Medical Sciences, Tehran, Iran. *; d *Nephrology and Urology Research Center, Baqiyatallah University of Medical Sciences, Tehran, Iran.*

**Keywords:** Kidney transplantation, N-acetylcysteine, Oxidative stress, Antioxidants, Hemodialysis, Graft function

## Abstract

N-acetylcysteine (NAC) is a potent antioxidant that acts through regenerating glutathione stores and scavenging oxygen-free radicals. This study assesses the short-term effects of NAC in cadaveric kidney transplant (KT) recipients. A double blind, randomized, placebo controlled trial was designed and patients were randomly assigned to receive either NAC or placebo. Glutathione peroxidase (GPX) activity in erythrocytes and serum malondialdehyde (MDA) levels were measured and serum creatinine levels and estimated glomerular filtration rate (eGFR) determined in the early phase after transplantation, were also compared between two study groups. Thirty-seven males and 20 females, with mean ± SD age of 44.6 ± 12.4 years completed the study. Significant difference (*P* = 0.02) was seen between GPX activity reduction in the placebo group, and that of the NAC group and on the levels of MDA there was no significant difference between two study groups (*P* = 0.53). Significant improvement in immediate graft function (IGF), (68% versus 40%, *P* = 0.05) and the first week eGFR were observed in the NAC group compared to the placebo group (52.46 ± 2.77 versus 38.75 ± 19.67 mL/min/1.73 m^2^, *P* = 0.02). It seems that the protective mechanisms of NAC, other than its antioxidant properties, can be favorable in KT patients.

## Introduction

Pre and post-transplant conditions can predispose the allograft to oxidative stress (OS) mediated injury which adversely affect the outcomes of transplantation (1). Glutathione peroxidase (GPX) is the primary and potent cellular defense system of erythrocytes against lipid peroxidation. It is shown that this enzyme is more potent than catalase and other antioxidant enzymes in protecting cells from oxidative stress (2). Antioxidant activity is reduced in patients with chronic kidney disease (CKD) (3, 4), especially in hemodialyzed (HD) patients (5, 6). Contact of blood with dialysis membrane triggers defense mechanism in erythrocytes and leads to reduced GPX activity (5, 7) which can be exacerbated by longer duration of HD (8). In addition, increase in OS, measured by lipid peroxidation products such as malondialdehyde (MDA), has been reported in HD patients (9).

After kidney transplantation (KT), OS can contribute to ischemia-reperfusion (I/R) injuries and graft dysfunction, which is more prevalent in deceased than living donor KT (1). I/R has been reported to contribute to the reduction of kidney glutathione stores that is an important intracellular antioxidant and also a substrate for GPX (10). Increased MDA can indicate the I/R stress of grafts and measuring its level on the first day after transplantation is shown to be a useful marker for early prediction of graft function (11). Furthermore, immunosuppressive therapy especially with cyclosporine represents an additional detrimental effect on erythrocyte GPX activity and enhances renal lipid peroxidation (11, 12). 

Reduction in the activity of GPX (13) and increase in MDA levels in KT patients show that free radical generation exceeds antioxidant defense (14), particularly during the first week after KT (15, 16). To counteract with the deleterious effects of lipid peroxidation, a highly effective antioxidant system is necessary to reduce the production of ROS and the damage from OS after kidney graft I/R injury (17). Several studies have been conducted to assess the effects of antioxidants, such as N-acetylcysteine (NAC), on OS status during the early phase after KT. NAC is a potent and safe antioxidant in kidney transplant recipients (18). Experimental models on I/R show that NAC can replenish renal glutathione levels 24 h after I/R and attenuate OS (10). It also improves the kidney function and reduces renal interstitial inﬂammation through decreasing infiltration of macrophages and lymphocytes (10, 19 and 20). These renoprotective effects and improvement in renal perfusion of graft by NAC are also reported in human studies (21-24).

The aim of the present study is to assess the effects of NAC on renal function and biomarkers of OS in cadaveric-donor renal transplant recipients, who had been on maintenance hemodialysis prior to transplantation. Comparing to previous studies (23, 25), our investigation considers most exposed patients to OS who had been on maintenance HD, received graft from cadaveric donor and were administered cyclosporine-based immunosuppression post transplantation.

## Experimental

A double blind, randomized, placebo-controlled study was conducted on adult recipients of cadaveric renal transplantation who had been on maintenance hemodialysis. The research was approved by the ethics committee of Shahid Beheshti University of Medical Sciences, and registered in the Iranian Registry of Clinical Trials (www.irct.ir, registration number: IRCT2016081827346N4). Written informed consent was obtained from all patients. Participants, care providers and those who assessed outcomes were blinded to the study groups.

*Study population and sample collection*

Eligible patients referring to the Shahid Labbafinejad and Baqiyatallah hospitals entered the study during the September 2015 to May 2016. A simple randomization was conducted and patients were divided into two groups to receive NAC or placebo with the cyclosporine-based immunosuppression protocol. Methylprednisolone 200 mg, IV infusion was administered before operation. Prednisolone 2 mg/kg (maximum dose120 mg/day), PO, was started on the first day post transplantation and tapered to reach a dose of 5-7.5 mg/day in three months. Mycophenolate mofetil (1.0 g given orally twice a day) was initiated before transplantation and continued postoperatively. Cyclosporine was administered twice daily with an initial dose of 7 mg/kg/day and was reduced to 6 mg/kg/day at the end of the third day, followed by drug level monitoring. Target cyclosporine trough level (C_0_) in our center is 150-300 ng/mL for the first 3 months after transplantation.

One dose of 600 mg NAC was administered before transplantation followed by twice daily of the same dose up to the fifth day after transplantation. 

Fasting venous blood samples were taken from all participants at baseline (pre-transplant) and on the first and fifth days after transplantation to measure the serum levels of MDA and erythrocyte activity of GPX. 

Whole blood samples were immediately frozen and stored at -70 °C until the date of analysis for GPX measurements. GPX activity was assayed in the hemolysate of whole blood (obtained by thawing of frozen specimens) at 340 nm using Randox commercial kits (made in the United Kingdom). The activity of erythrocyte antioxidant enzyme was expressed as units of enzyme per gram of hemoglobin.

To measure the level of MDA, a gel-containing tube without anticoagulant was used. Then, the serum was separated using centrifuge for about 5 min at 3000 rpm. The extracted serum was transferred to microtubes labeled with identifier code and was kept at -70 °C until the analysis time. The serum MDA level was measured using the method described by Satoh (26) through thiobarbituric acid (TBA) reaction and separation on HPLC. UV detection was performed at 532 nm.

*Inclusion and exclusion criteria*

The patients included in the study were adult recipients (aged between 18 to 75 years) of deceased donor kidney transplant who were on maintenance dialysis prior to transplantation.

We excluded preemptive kidney transplantation (transplantation prior to initiation of dialysis) which is reported to have superior outcomes in graft and patient survival (27). In addition, multi-organ transplants, second transplantation, history of using NAC within the month prior to operation, and history of sensitivity to sulfa drugs were considered as exclusion criteria in this study.

*Study outcomes*

The primary outcomes of the study were to determine the GPX activity and MDA levels of NAC recipients during early phase after transplantation by blood sampling 2 h prior to, and on day 1 (only for MDA assessment) and day 5 of transplantation. In addition, the rate of decrease in serum creatinine within 48 h of transplantation (creatinine reduction ratio), and the graft function at the end of the first and second weeks, as well as at the end of the first month post transplantation were compared.

*Statistical analysis*

Statistical analysis was performed using SPSS software (Statistical Package for the Social Sciences, version 21.0; SPSS Inc., Chicago, Illinois, USA). The results are expressed as mean ± standard deviations (SD) for variables with normal distribution, and median (IQR; interquartile range) if distribution of a variable was not normal. The Kolmogorov–Smirnov test was used to assess the normal distribution of all analyzed data. Comparisons were performed using the unpaired Student’s *t*-test for variables with normal distribution and Mann–Whitney U-test for variables with skewed distribution. Chi-square and Fisher’s exact tests were utilized in the analyses of nominal variables. The repeated measures ANOVA was used for the analysis of MDA measured at baseline, the 1^st^ and 5^th^ days after KT. *P*-values less than 0.05 were considered as statistically significant. Estimated glomerular filtration rate (eGFR) was calculated using the Modification of Diet in Renal Disease (MDRD) study equation. Creatinine reduction ratio (CRR2) within 48 h after transplant was calculated by dividing the difference of day 1 and 2 levels by the day 1 value (CRR2 (%) = ([C1−C2] × 100)/C1). The patients in either of the two groups with CRR2 > 30% were considered to have immediate graft function after transplantation (28).

*Sample size estimation*

Sample size was determined based on the GPX activity and MDA levels as the main outcomes of the study. Minimum sample size of 15 and 12 (in each study group) were estimated for the GPX activity and MDA levels, respectively, based on the power of 90%, and α = 0.05. 

## Results

**Figure 1:**
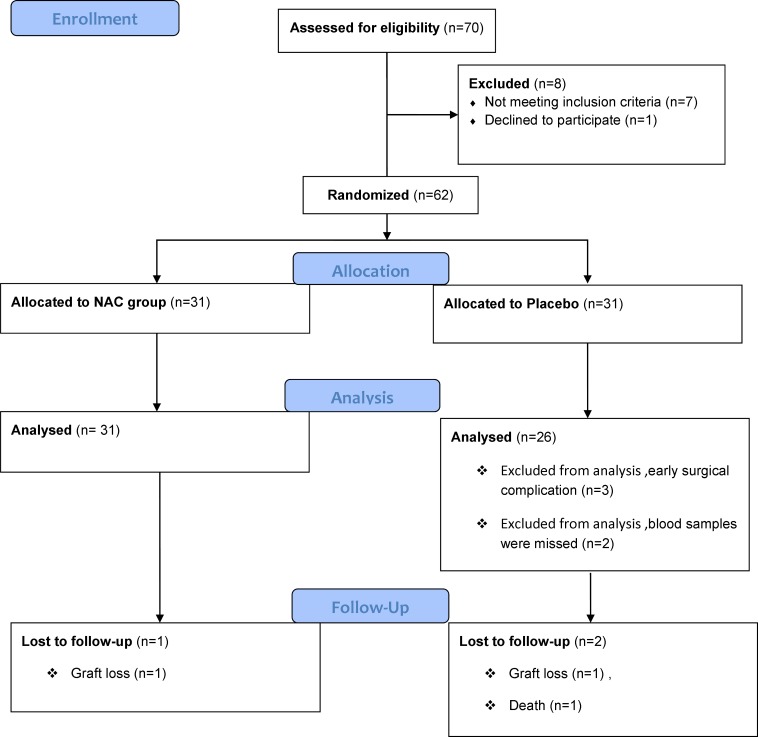
Study flowchart

From 70 recipients of cadaveric renal transplantation who were assessed for eligibility to enter the study, 62 patients fulfilled the criteria (31 in the NAC and 31 in the placebo group). Five patients in the placebo group were then excluded due to early surgical complication and missed blood samples. Thus, the analysis was performed on 57 patients, 31 in the NAC and 26 in the placebo groups. The study flowchart is shown in Figure 1.

**Table 1:** Demographic and clinical characteristics of deceased kidney donors.

	**Placebo group**	**NAC group**	***p*** **-value**
Age, years (Mean ± SD)	42.19 ± 15.23	38.52 ± 14.26	0.35
Gender, n (%) Female Male	3 (11.5) 23 (88.5)	10 (32.0) 21 (68.0)	0.11
BMI, kg/m^2 ^, (Mean ± SD)	25.27 ± 3.57	25.43 ± 4.53	0.60
eGFR, mL/min/1.73 m2 (Mean ± SD)	75.92 ± 24.99	73.77 ± 28.75	0.60

**Table 2:** Demographic and clinical characteristics of recipients

	**Placebo group**	**NAC group**	***p*** **-value**
Age, years (Mean ± SD)	46.65 ± 12.31	42.87 ± 12.37	0.25
Gender, n (%) Female Male	10 (38.5) 16 (61.5)	10 (32.0) 21 (6.08)	0.78
BMI , kg/m^2 ^, (Mean ± SD)	25.42 ± 4.72	24.04 ± 3.88	0.23
Time on dialysis before transplantation, month (Mean ± SD)	17.02 ± 10.52	14.3 ± 14.49	0.14
Baseline serum creatinine, mg/dL, (Mean ± SD)	6.97 ± 1.99	7.35 ± 2.47	0.53
Hemoglobin, g/dL, (Mean ± SD)	10.50 ± 1.90	11.26 ± 2.08	0.20
Underlying disease, n (%)			
Diabetes mellitus Hypertension Glomerulonephritis Polycystic kidney disease Renal stone Others	6 (23) 7 (27) 0 2 (8) 3 (11) 8 (31)	9 (29) 6 (19) 5 (16) 3 (10) 0 8 (26)	0.31

**Table 3:** Baseline and follow-up measurements of glutathione peroxidase and malondialdehyde during study intervention

	**Placebo group**	**NAC group**	***p*** **-value**
GPX0 (U/g Hg), Mean ± SD Median (interquartile range)	20.71 ± 17.89 16.82 (12.60-18.50)	14.61 ± 4.88 14.64 (10.09-17.87)	0.32
GPX5 (U/g Hg), Mean ± SD Median (interquartile range)	15.93 ± 11.64 14.72 (10.51-17.87)	16.59 ± 7.78 15.14 (12.19-21.23)	0.58
GPX5-GPX0 (difference), Mean ± SD Median (interquartile range)	-4.80 ± 10.06 -1.16 (-8.50 –1.25)	2.01 ± 6.73 +1.81 (-2.50 –8.00)	0.02
MDA0 (nmol/mL), Mean ± SD Median (interquartile range)	3.54 ± 1.85 3.40 (1.80-5.05)	4.34 ± 2.26 3.60 (2.70-6.95)	0.28
MDA1 (nmol/mL), Mean ± SD Median (interquartile range)	4.31 ± 2.28 3.95 (2.22-5.85)	4.24 ± 2.39 3.65 (2.77-6.30)	0.85
MDA5 (nmol/mL), Mean ± SD Median (interquartile range)	4.37 ± 2.41 4.0 (2.60-6.27)	4.74 ± 1.54 4.70 (3.80-5.60)	0.49

* GPX 0: pre-transplant (baseline) glutathione peroxidase activity; GPX 5: fifth day glutathione peroxidase activity, MDA0: pretransplant malondialdehyde; MDA1: day 1 malondialdehyde; MDA5: day malondialdehyde level; NAC: N-acetylcysteine.

**Table 4.** Estimated glomerular filtration rate (mean ± SD) in two study groups during the first month after transplantation

**eGFR (mL/min.1.73 m** ^2^ **)**	**Placebo group**	**NAC group**	***p*** **-value**
7^th^ day	38.75 ± 19.67	52.46 ± 2.77	0.02
14^th^ day	46.58 ± 19.68	55.66 ± 19.77	0.07
30^th^ day	53.50 ± 14.07	58.30 ± 18.53	0.30

No statistically significant differences were observed between two groups in terms of demographic data and baseline clinical characteristics. Tables 1 and 2 show all relevant demographic and clinical characteristics of the donors and recipients.

In both groups, approximately 50% of patients were dialyzed within 24 h prior to transplantation (51.6% in NAC group versus 50% in placebo group). The percentage of patients dialyzed between 24 and 48 h prior to transplantation were 29.0% and 42.3% in the NAC and placebo groups, respectively. The dialysis of the remaining patients (19.4% in NAC and 7.7% in placebo group) took place afore 48 h before transplantation (*P* = 0.16).

For all participants, panel reactive antibody (PRA) was negative and the cold ischemia time was less than 6 h. Cyclosporine trough levels (C_0_) were 241.35 ± 99.60 ng/mL in NAC group and 245.10 ± 87.56 ng/mL in placebo group (*P* = 0.92) during the immediate postoperative period. None of the patients experienced any adverse effect from NAC or placebo during the study period.

The baseline and day 5 erythrocyte glutathione peroxidase activities (GPX0 and GPX5) are given in Table 3. The results show no statistical differences between two groups for both GPX0 and GPX5 (*P* = 0.32 and *P* = 0.58 respectively). However, the median (interquartile range) of differences between day 5 and baseline levels of GPX were -1.16 (-8.5 – 1.25) for the placebo group and +1.81 (-2.5 – 8) for the NAC group (*P* = 0.02), which shows that the relative improvement in the activity of enzyme in the NAC group is statistically significant. 

The analysis of MDA levels is shown in Table 3. The baseline levels (MDA0) were not statistically different between two groups (*P* = 0.28). Day 1 results (MDA1) increased 0.60 ± 1.97 nmol/mL from baseline in the placebo group and decreased 1.64 ± 2.93 nmol/mL in the NAC group, however the difference was not statistically significant (*P* = 0.29). The increase in the MDA level after five days (MDA5 – MDA0) has a mean of 0.83 ± 3.07 nmol/mL in the placebo and 0.5 ± 2.1 in the NAC groups. Similarly, it was not statistically different between two groups (*P* = 0.65). In addition, the repeated measures analysis of variance (ANOVA) on three levels of MDA for baseline, days 1 and 5 showed no significant difference between the NAC and placebo groups (*P* = 0.53).

The calculated eGFR at the end of the first week after KT showed significant improvement in the NAC group compared to that of the placebo group (*P* = 0.02). In addition, immediate graft function was observed in 40% of patients in the placebo group and 68% in the NAC group (*P* = 0.05). 

The median (interquartile range) length of hospital stay was 12.0 (10.0–16.5) days in the placebo and 15.0 (12.0–21.0) days in the NAC group (*P* = 0.08).

The results of mean eGFR within the first month follow-up are given in Table 4. It confirms a faster recovery of graft function in the NAC group. One case of graft loss in each of the NAC and placebo groups, as well as two cases of acute rejection and one death in the placebo group were recorded during the first month follow-up.

## Discussion

Our results showed that treatment with NAC could significantly increase eGFR during the first week after KT in hemodialyzed patients. The repeated measures ANOVA on MDA levels showed no significant change for this biomarker in the NAC group. This could be a result of the fact that patients entered into this study were exposed to several risk factors for OS including maintenance HD, cadaveric renal transplantation and cyclosporine-based immunosuppression. Perhaps, dose and duration of NAC administration were not large enough to lead to statistically significant improvements in OS status of the intervention group. 

The renal function during the first week after transplantation improved significantly in the NAC group (*P* = 0.02). Also, the number of patients with immediate graft function was significantly higher in the NAC group (*P* = 0.05), which shows that administration of NAC can lead to a faster reduction rate in serum creatinine within the first 48 h post-KT. In a similar study on cadaveric renal transplant patients receiving the same daily dose of NAC for seven days, reported by Danilovic *et al.,* a faster allograft function recovery with NAC was also observed throughout the 3-month follow-up (23). 

As shown in Table 3, despite the administration of NAC, the MDA levels measured during the study remained high, which shows that the antioxidant status was not sufficient to protect cells during oxidant exposure. Petronilho *et al.* suggested that some mechanisms other than antioxidant properties could be involved in the nephroprotection provided by NAC and that its additional protective effects may be resulted from reduction of NF-kB activation and interstitial inflammation (29). Likewise NAC has endothelium-dependent vasodilatory effect and can improve renal perfusion through stabilizing NO and reducing angiotensin II (17). 

The results from this study and the work conducted by Danilovic *et al.* approve that longer duration of NAC administration may be more effective in encountering OS through strengthening the antioxidant defense system. In addition, since the antioxidant effect of NAC has shown to be dose-dependent (30), higher doses of oral NAC *e.g.* 1200 mg/day can be studied for lowering OS in HD patients who receive KT. 

## Conclusions

This study has assessed the effect of oral NAC administration, as a safe, inexpensive, and well-tolerated drug, in reducing OS in HD patients who received cadaveric-donor KT. The effects were studied through measurement of the erythrocyte GPX and MDA levels in the early phase after transplantation. In addition, serum creatinine and eGFR were assessed. The results showed that NAC can improve allograft function in the first week post-KT with no significant effect on the OS biomarkers. These results suggest that mechanisms other than scavenging oxygen-derived free radicals are involved in protecting against I/R injuries of graft in cadaveric kidney transplantation. Although, NAC did not decrease the biomarker of OS, it has several clinical benefits which support its use for KT patients. Future studies may consider higher doses and longer period of NAC administration.
